# Randomized Clinical Trial with e-MotionalTraining^®^ 1.0 for Social Cognition Rehabilitation in Schizophrenia

**DOI:** 10.3389/fpsyt.2018.00040

**Published:** 2018-02-26

**Authors:** Yolanda Maroño Souto, Miriam Vázquez Campo, Francisco Díaz Llenderrozas, Marina Rodríguez Álvarez, Raimundo Mateos, Alejandro García Caballero

**Affiliations:** ^1^Department of Psychobiology and Clinical Psychology, Psychology School, University of Santiago de Compostela, Santiago de Compostela, Spain; ^2^Department of Psychiatry, Instituto Biomédico Galicia Sur, Centro de Investigación Biomédica en Red de Salud Mental (CIBERSAM), Complexo Hospitalario Universitario de Ourense, Ourense, Spain; ^3^Complexo Hospitalario Universitario de Santiago de Compostela, Santiago de Compostela, Spain; ^4^Department of Psychiatry, School of Medicine, University of Santiago de Compostela, Santiago de Compostela, Spain

**Keywords:** cognition, emotional adjustment, theory of mind, schizophrenia, emocional perception

## Abstract

**Background:**

Schizophrenia patients present deficits in social cognition (SC), emotion and social perception, theory of mind (ToM), and attributional style. This study tested the efficacy, in real clinical conditions, of a online self-training program in SC, e-Motional Training^®^, in comparison with treatment as usual.

**Method:**

A randomized single-blinded multicenter clinical trial was conducted with 60 schizophrenia stable outpatients. All patients (control and intervention) were treated with drug therapy, case management, and individual and group psychotherapy (not focused on SC). Intervention group was treated with e-Motional Training^®^, an online program devised for SC rehabilitation.

**Statistical analysis:**

A descriptive analysis and parametric/non-parametric tests were used to compare both groups at baseline. Analysis of covariance was used to compared post–pre changes in SC between the two interventions. If the group effect was significant, follow-up univariate test (*t*-test for dependent samples) was carried out in each group to verify whether the effect was due to improvement in the intervention group or deterioration in the control group. We considered statistically significant differences with *P* < 0.05.

**Results:**

Significant improvements were obtained in the intervention group in emotion recognition and most ToM variables in comparison with the control group.

**Discussion:**

*e-Motional Training*^®^ seems to be a promising online training tool for SC deficits in schizophrenia, covering the lack of similar intervention instruments in our community.

## Background

Social cognition (SC) is defined as the mental operations that underpin perceiving, interpreting, and generating responses during social interactions, including the intentions, dispositions, and behaviors of others (1). The Social Cognition Psychometric Evaluation study identified four core domains of SC, namely emotion recognition (ER), social perception, theory of mind (ToM)/mental state attribution, and attributional style (AS)/bias (2).

In schizophrenia, negative symptoms have been associated with poor performance in SC (3). In particular, individuals with schizophrenia show deficiencies in ER compared with non-clinical participants (4, 5), and these difficulties are significantly associated with symptom severity (6). These limitations are primarily manifested in the identification of negative valence emotions, especially the emotion of fear (7–10). Longitudinal studies have shown that these difficulties are stable over the course of the disease (11, 12) although there is evidence that individuals in the remission phase perform better on ER tests than individuals who are in the acute phase of the disorder (6). These difficulties are also considered to have a moderate association with social functioning of hospitalized patients (13) in comparison with outpatients (14).

Moreover, difficulties in ToM have been associated with negative symptoms, passivity, behavioral disorders, and paranoid symptoms (3, 15, 16). Studies have found that greater hostile attributions (e.g., increased tendency to report guilt/hostility/aggression in response to ambiguous social situations) correlate with higher levels of positive symptoms, anxiety, depression, and general emotional discomfort (17, 18).

In schizophrenia, these difficulties are associated with poorer social functioning (19), fewer social relationships, and poorer quality of life (5, 20). Various research studies have found that SC serves as a mediator between neurocognition and functional results (21) and determines the quality of interpersonal interactions, which facilitates the enjoyment of recreational activities for individuals with schizophrenia (22–25). SC is considered a predictor of social functioning even more relevant than neurocognition (19). However, these difficulties are not restricted to schizophrenia but are also observed in other severe mental disorders (26–28).

Patients with schizophrenia often report these difficulties. Therefore, there is a urgent need to find new treatment strategies to enable individuals with schizophrenia to improve these skills (29), given that drug treatments (typical and atypical antipsychotics) generally only have a marginal impact on the domains that constitute SC and social functioning (30). Conversely, there is evidence that SC in schizophrenia can be improved through psychosocial intervention (29, 31–34).

In view of the significant impact of social cognitive deficits on daily functioning, many interventions have been developed over the past decade to ameliorate social cognitive deficits. Some interventions using virtual reality, cognitive behavioral techniques, and errorless learning in social skills training show positive results in social functioning, but without specifically targeting SC (35–38). Targeted interventions hold much promise for improving SC, particularly ER and ToM. Improvement in ER has been reported, particularly in facial affect recognition. Most of these targeted interventions, such as Training of Affect Recognition (39), Attention Shaping, or MicroExpression Training Tool (40), focus primarily on training affect recognition with good outcomes. ToM is the second most commonly targeted domain, with Mental-State Reasoning Training for Social Cognitive Impairment (SoCog-MSRT) (41), Mary Eddie Bill (MEB) (42), Emotion and ToM Imitation (43), and Theory of Mind Intervention (44) developed to provide effective in-depth training, but with contradictory results in this domain (45). AS is only specifically targeted in SoCog-MSRT and MEB. Social perception and AS appear to be more difficult to measure and train, as evidenced by a meta-analysis that showed no significant effects on these two domains after social cognitive training (46).

Besides video clips, cartoon comic strips, and photographs, computerized online social cognitive games and virtual reality have recently been utilized with high patient satisfaction (36, 37, 47). Specifically, virtual reality has been used for social skills training, but its application has not been yet oriented toward SC training.

*e-Motional Training*^®^ 1.0 (ET) allows online self-training and stores the data of each individual session. ET is designed following the basic principles of neuropsychological rehabilitation in this domain (48–50). The program aims to deliver realistic and natural but attractive exercises of short duration without irrelevant stimuli or distractions, while offering continuous feedback. ER tasks are designed with increasing difficulty, starting with tutorials, following with eyes and mouths recognition and finally scaling to microexpression training. An animated short film with 33 scenes is the vehicle for ToM, social perception, and AS stories. After each scene, a series of questions including ToM, AS, and control questions are posed. When the answer is incorrect, the patient receives metacognitive suggestions, which lead the user to think about the situation from a different perspective or prompts the user to pay attention to specific aspects of the film.

The program was composed of 12 1-h sessions (the minimum number of face-to-face sessions reported in previous studies).

Our hypothesis was that intervention with treatment as usual (TAU) + ET results in greater improvements in the main domains of SC and the measures of social functioning compared with TAU.

The aim of this study is therefore to assess the possible effects of a new SC training program, *e-Motional Training*^®^ 1.0 (ET), in ER, ToM, AS, and social functioning.

## Method

A randomized, multicenter, single-blind clinical trial was performed. Sixty patients with schizophrenia were recruited in Psychiatric Day Hospitals at Ourense, Coruña and Vigo and in Associations of Persons and Families with Mental Illness at Vigo, Santiago de Compostela, Coruña and Ourense. After recruitment, the sample was randomized in each center into two balanced groups.

### Inclusion and Exclusion Criteria

We included patients who voluntarily agreed to participate in the study, aged 18–50 years with a diagnosis of schizophrenia (DSM-IV TR), who were clinically stable (no acute psychotic symptoms and not hospitalized during the last 3 months), and who had no comorbidity with other psychiatric or neurological diseases (International Neuropsychiatric Interview-MINI) and excluding current substance abuse (except nicotine).

### Treatment Conditions

#### Control Group (TAU)

All patients received drug therapy, case management, and individual and group psychotherapy not focused on social cognitive rehabilitation.

#### Intervention Group (TAU + ET)

The intervention group received the same intervention of control group plus 12 sessions (1 h per week) with ET^®^. All participants in the intervention group completed the same number of sessions. To start the intervention, the patient accessed the website www.e-motionaltraining.com (version 1.0) and registered with a username and password. The first four meetings (1 h each session) were dedicated to recognizing facial emotions. This section included a pretest and posttest, tutorials, and scaling minigames starting with eyes and mouths and finally microexpression (<250 ms) training. The next eight sessions (1 h each) include watching a short, interactive animated cartoon in which a couple invites their friends to their home for a party. As the story unfolds, instances of miscommunication occur among the actors, causing various emotions and mental conditions such as anger, affection, appreciation, and jealousy. After each scene, the user is queried about what happened, with questions about ToM (interpreting irony, insinuations, faux pas, second-order false beliefs, etc.), social perception (interpretation and analysis of the social situation through the visual content of each scene), and AS (the individuals’ attributions to the events, and questions such as, “What kind of thinking would result in Cristina getting better results in this situation?”), as well as control questions. The game provides user feedback and, in the event of errors, can display a hyperlink with information and metacognitive strategies, whose objective is to help users understand the scene that they just watched.

Supervision of the ET group was conducted by the center’s staff as a routine activity, and evaluators were blind to the assignment. No help or guidance regarding social cognitive issues was given, and only advice regarding computer use was provided.

### Measurements

#### Symptoms and Cognitive Ability

##### Positive and Negative Symptom Scale (PANSS)

Positive and Negative Symptom Scale assesses positive and negative symptom severity (51). The scale consists of 30 items (symptoms) that are scored from 1 (absent) to 7 (extreme). The scale has three subscales: *positive* (PANSS-P), *negative* (PANSS-N), and *general psychopathology* (PANSS-GP).

##### Kaufman Brief Intelligence Test (K-BIT)

Kaufman Brief Intelligence Test provides a verbal intelligence quotient (IQ), a non-verbal IQ, and a compound IQ that summarizes the total performance on the test (52).

#### Social Cognition

##### Ekman 60 Faces Test

The test contains 60 photographs of faces with expressions of the 6 basic emotions: anger, disgust, sadness, fear, surprise, and happiness (53). An overall score of 60 indicates the best possible performance, and each basic emotion also has a maximum score of 10 points.

##### Hinting Task

Ten stories are presented to the patient who must infer the characters actual intention when using indirect speech (54). The total score on the test ranges from 0 to 20 (55).

##### Recognition of Faux Pas

The participant must recognize the embarrassing situations in the 10 *faux pas’* stories, while correctly rejecting misinterpretation of the 10 control situations (56). The test provides scores for five variables: *faux pas* detection, understanding inappropriateness, intentions, and belief and empathy (57).

##### F. Happé’s Strange Stories

F. Happé’s Strange Stories include stories containing irony and white lies utterances (58). In each of the stories, the character says something that should not be interpreted literally. The participant is asked to explain why the characters said what they said.

##### Movie for the Assessment of Social Cognition (MASC)

A short film is shown to the participant who must answer a series of questions regarding the ToM and emotional content depicted in social interactions (59).

##### Ambiguous Intentions Hostility Questionnaire (AIHQ)

The AIHQ is an AS questionnaire to measure the biases of hostility perception, composite blame, and aggressive response (60). The AIHQ is composed of 15 hypothetical negative situations. Each situation was varied in intentionality: five scenarios are accidental (e.g., “You’re dancing at a club and someone bumps into you from behind.”), five scenarios are ambiguous (e.g., “You walk past a bunch of teenagers at a mall and your hear them start to laugh.”), and five scenarios are intentional (e.g., “Your neighbors are playing loud music. You knock on the door and ask them to turn it down. Fifteen minutes later, the music is loud again.”). First, participants are prompted to imagine the scenario happening to them. Then, they are asked to write down what is the reason they think that other person (or persons) acted that way. The AIHQ yielded hostility perception and aggressive response bias scores and a composite blame bias score. The scales for the hostility perception and aggressive response indices were rated by rater from 1 (“not at all hostile”) to 5 (“very hostile”) and 1 (“not at all aggressive”) to 5 (“very aggressive”), respectively. The composite blame score (range, 1–5.3) is an average score of subjects’ ratings of intent (range, 1–6; rating about the degree to which the other person committed the act on purpose), anger (range, 1–5; rating about how angry the situation would make subject feel), and blame (range, 1–5; rating about how much subjects blame the other person for the outcome).

#### Emotional Intelligence

##### Mayer-Salovey-Caruso Emotional Intelligence Test (MSCEIT)

This test is composed of 141 items and provides a score for emotional intelligence (EIQ), which in turn can be divided into two domains: experiential (EEIQ) and strategic emotional intelligence (SEIQ) (61). The test also provides scores for four areas of emotional intelligence: the ability to perceive emotions accurately (PEIQ), using emotions to facilitate thought (emotional facilitation, FEIQ), understanding emotions (UEIQ), and managing emotions (MEIQ).

#### Social Functioning

##### Social Functioning Scale (SFS)

This scale is specifically designed to assess the social functioning of individuals with schizophrenia (62). The scale consists of seven subscales: social isolation/interaction, interpersonal communication, independence-execution, independence-competence, free time, prosocial activities, and employment/occupation. We applied the self-reported version (SFS-SR).

### Sample Size

In our pilot study (63), the measure with most reduced differences pre–post intervention was Happé’s Strange Stories with an initial mean (±SD) of 8.20 (±3.58) that increased to 11.20 (±4.68) after intervention. By using these measures for a power of 80% and a confidence level of 95%, the required sample size, assuming 5% of losses, was 30 patients in each group.

### Ethical Aspects

This study has been carried out in accordance with national and European legislation on clinical research, following international ethical recommendations, the Declaration of Helsinki, and the Council of Europe with regard to the Convention on Human Rights and Biomedicine. The study has complied at all times with the requirements established in the Spanish legislation in the field of biomedical research, personal data protection, and bioethics. This study was approved by the local ethics committee (Comité de Etica e Investigación Clínica de Galicia) (Registration code: 2014/459) and registered in an international RCT database (BioMed Center: ISRCTN83459317).

### Statistical Analysis

Quantitative Gaussian variables were described by mean, SD, and not Gaussian variables as median (range). The qualitative variables were described by frequencies and percentages (%). Parametric/non-parametric tests (Chi square for categorical variables and Student’s *t*-test and Mann–Whitney *U* test for continuous variables) were used to compare both groups at baseline.

We compared post–pre changes in SC between the two interventions (TAU + e-Motional Training^®^ vs. TAU) with an analysis of covariance (ANCOVA), entering the change scores on each test (Ekman, Faux Pas, Happé, Hinting, MASC, MSCEIT, and AIHQ) as the dependent variable, treatment as the fixed group effect, and K-BIT score as the covariate.

If the group effect was significant, follow-up univariate test (*t*-test for dependent samples) was carried out in each group to verify whether the effect was due to improvement in the intervention group or deterioration in the control group.

We considered statistically significant differences with *P* < 0.05. The sample size was calculated using the Epidat 4.1, and the analyses were performed with SPSS 22.0 and R (http://www.r-project.org).

## Results

**Figure 1:**
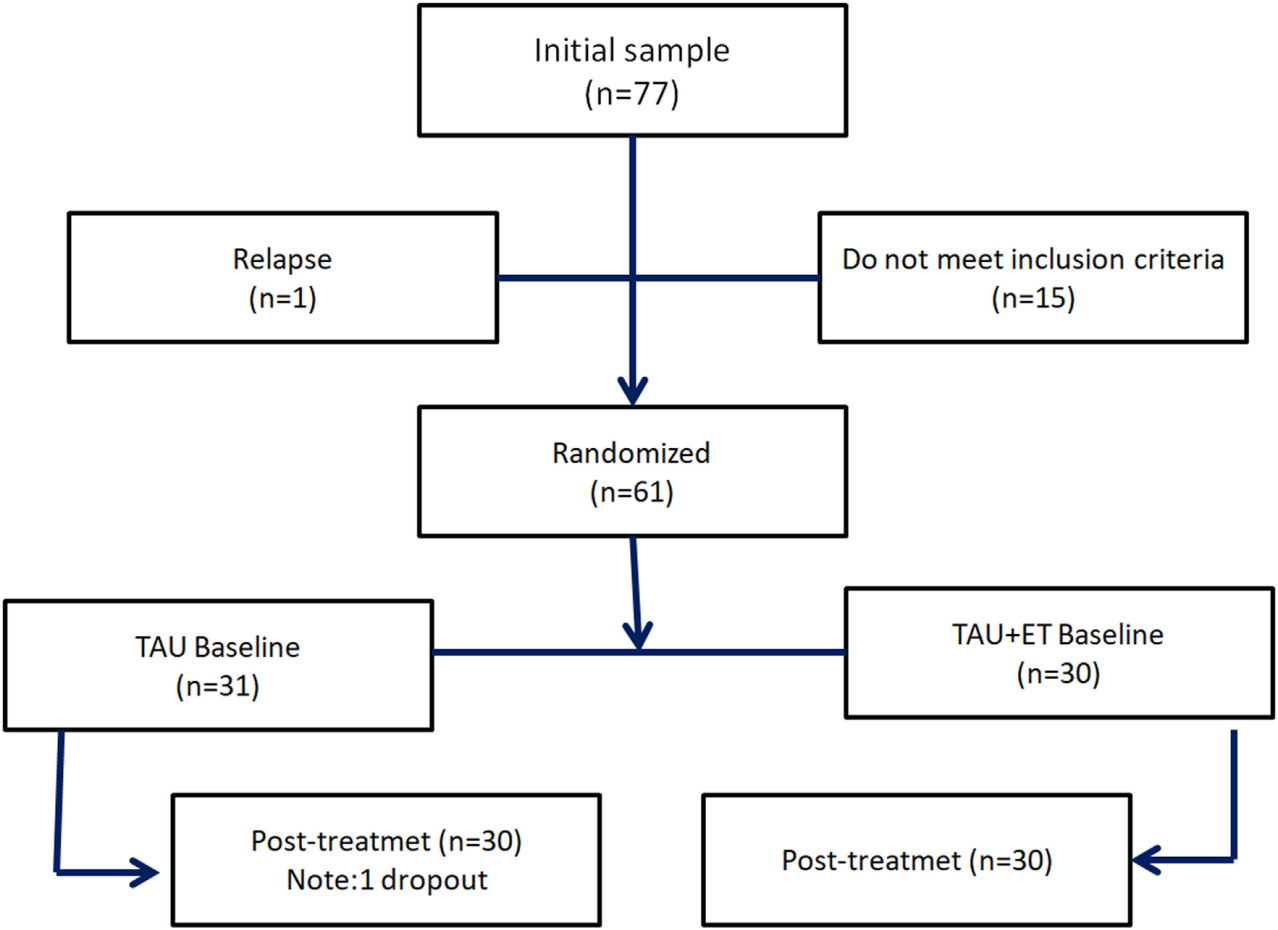
Flowchart.

A total of 77 participants were selected, 15 patients did not meet inclusion criteria, and 1 suffered a relapse prior to randomization. Finally, 61 patients were assigned to the control group (TAU) or to the intervention group (TAU + ET) between January and November 2015 (Figure 1). Prior to retest one patient in the control group abandoned the study and was excluded for further analysis.

Most of the patients recruited were men 47 (78.3%), with a mean (±SD) global age of 39.17 years (±7.03).

There were no significant differences between the two groups at baseline in sociodemographic variables (age, gender, and education) compound IQ (*P* = 0.385) and non-verbal IQ (*P* = 0.143) measured with K-BIT. However, significant differences were observed in the verbal IQ (*P* = 0.042) being the scores in both groups within the normality range (Table 1).

**Table 1:** Demographic and clinical characteristics of sample (*N* = 60).

	TAU	TAU + ET	Global	*P* value
**Gender, *n* (%)**				
Male	23 (76.7)	24 (80.0)	47 (78.3)	0.754
Female	7 (23.3)	6 (20.0)	13 (21.7)	

**Education, *n* (%)**				
Primary	15 (50.0)	11 (36.7)	26 (43.3)	0.297
Secondary	15 (50.0)	19 (63.3)	34 (56.07)	

**Continuous variables, mean (±SD)**				
Age (years)	39.87 (±6.12)	38.47 (±7.88)	39.17 (±7.03)	0.445^a^
Age at first hospitalization (years)	24.93 (±7.20)	23.48 (±7.20)	24.22 (±7.18)	0.443^a^
Medication dose (chlorpromazine)	634.82 (±513.01)	564.74 (±340.19)	599.78 (±433.00)	0.807^a^
Lifetime number of hospitalizations, median (range)	2 (0–11)	1 (0–12)	1 (0–12)	0.331^b^

**PANSS**				
PANSS-P	13.13 (±5.43)	15.83 (±6.74)	14.48 (±6.22)	0.093^a^
PANSS-N	18.60 (±8.11)	17.67 (±9.12)	18.13 (±8.57)	0.677^a^

**IQ (K-BIT)**				
Overall IQ	95.30 (±12.80)	101.60 (±37.30)	98.45 (±27.83)	0.385^a^
Verbal	95.47 (±20.28)	104.50 (±12.16)	99.98 (±17.19)	0.042^a^
Non-verbal	83.17 (±24.45)	91.20 (±16.76)	87.18 (±21.17)	0.143^a^

*Data are represented as *n* (%) for categorical variables, mean (±SD), and median (range). *P* value: Chi square test*.

*^a^Student’s t-test*.

*^b^Mann–Whitney U test*.

All participants were treated with antipsychotics, with a mean chlorpromazine dose 634.82 (±513.01) in control group and 564.74 (±340.19) in the intervention group. There were no significant differences between them (*P* = 0.807).

To demonstrate the existence of differences in SC variables after treatment, we compared post–pre changes between the two interventions (TAU + e-Motional Training^®^ vs. TAU) with an ANCOVA, entering the change scores on each test (Ekman, Faux Pas, Happé, Hinting, MASC, MSCEIT, and AIHQ) as the dependent variable, treatment as the fixed group effect, and K-Bit score as the covariate. ANCOVA results are displayed in Table 2. These results indicate that there were statistically significant differences in change scores between e-Motional Training^®^ and TAU group in Ekman’s (*F* = 48.805, *P* < 0.001) with a large effect size $\left(\eta_{p}^{2}=0.461\right)$, Faux Pas (*F* = 9.728; *P* = 0.003) with a large size effect $\left(\eta_{p}^{2}=0.146\right)$, Happé ToM (*F* = 9.447; *P* = 0.003) with a large effect size $\left(\eta_{p}^{2}=0.142\right)$, Hinting (*F* = 14.286; *P* < 0.001) with a large effect size $\left(\eta_{p}^{2}=0.200\right)$, MASC change score (*F* = 12.466; *P* = 0.001) with a large size effect $\left(\eta_{p}^{2}=0.179\right)$, and PANSS negative change score (*F* = 5.169; *P* = 0.027) with a moderate size effect $\left(\eta_{p}^{2}=0.083\right)$. No differences were found in Faux Pas and Happé control stories change scores nor in MSCEIT or PANSS positive change scores. Finally, regarding the Ambiguous Stories of AIHQ, only differences in aggressive bias (*F* = 4.405; *P* = 0.04) were significant with a moderate size effect $\left(\eta_{p}^{2}=0.072\right)$.

**Table 2:** Results in social cognition variables.

	TAU		TAU + ET		*F*	*P* value	Effect size $\eta_{p}^{2}$
	Mean (IC 95%)	SD	Mean (IC 95%)	SD			
Ekman pre	41.23 (38.66 to 43.80)	6.89	42.77 (40.76 to 44.77)	5.36			
Ekman post	41.03 (38.17 to 43.90)	7.68	50.57 (48.15 to 52.99)	6.48			
Ekman change	−0.20 (−1.87 to 1.47)	4.48	7.80 (6.12 to 9.48)	4.49	48.805	<0.001	0.461
Faux Pas HC pre	18.53 (17.86 to 19.21)	1.81	16.53 (15.19 to 17.88)	3.60			
Faux Pas HC post	18.47 (17.62 to 19.31)	2.27	17.60 (16.55 to 18.65)	2.80			
Faux Pas HC Change	−0.07 (−0.81 to 0.68)	2.00	1.07 (0.29 to 1.84)	2.08	4.022	0.050	0.066
Faux Pas pre	27.37 (21.50 to 33.23)	15.71	28.97 (23.72 to 34.21)	14.05			
Faux Pas post	28.47 (22.61 to 34.32)	15.69	37.10 (31.63 to 42.57)	14.64			
Faux Pas change	1.10 (−1.51 to 3.71)	6.98	8.13 (4.60 to 11.67)	9.47	9.728	0.003	0.146
Happè TOM pre	8.63 (7.10 to 10.17)	4.12	8.13 (6.86 to 9.40)	3.40			
Happè TOM post	9.20 (7.64 to 10.76)	4.17	10.80 (9.38 to 12.22)	3.81			
Happè TOM change	0.57 (−0.36 to 1.49)	2.47	2.67 (1.58 to 3.75)	2.90	9.447	0.003	0.142
Happè HC pre	9.07 (7.65 to 10.48)	3.79	8.77 (7.56 to 9.97)	3.22			
Happè HC post	10.00 (8.66 to 11.34)	3.59	10.60 (9.42 to 11.78)	3.16			
Happè HC change	0.93 (−0.07 to 1.94)	2.69	1.83 (0.75 to 2.92)	2.90	1.703	0.197	0.029
Hinting pre	15.23 (14.12 to 16.35)	2.98	13.60 (11.80 to 15.40)	4.83			
Hinting post	15.60 (14.43 to 16.77)	3.14	16.63 (15.15 to 18.12)	3.98			
Hinting change	0.37 (−0.32 to 1.06)	1.85	3.03 (1.79 to 4.28)	3.34	14.286	<0.001	0.200
MASC pre	21.97 (19.71 to 24.23)	6.05	23.17 (21.47 to 24.87)	4.55			
MASC post	21.97 (19.81 to 24.12)	5.77	26.23 (24.28 to 28.19)	5.23			
MASC change	0.00 (−1.22 to 1.22)	3.26	3.07 (1.84 to 4.30)	3.29	12.466	0.001	0.179
MSCIT pre	91.80 (86.76 to 96.84)	13.50	94.83 (90.07 to 99.58)	12.50			
MSCIT post	90.80 (86.06 to 95.54)	12.69	95.60 (91.14 to 100.06)	11.94			
MSCIT change	−1.00 (−3.95 to 1.95)	7.91	0.03 (−2.55 to 2.62)	6.80	0.315	0.577	0.006
PANSS-P pre	13.13 (11.11 to 15.16)	5.42	15.83 (13.32 to 18.35)	6.74			
PANSS-P post	12.03 (10.18 to 13.88)	4.95	14.77 (12.28 to 17.25)	6.65			
PANSS-P change	−1.10 (−1.63 to−0.57)	1.42	−1.07 (−2.56 to 0.42)	3.99	0.002	0.967	<0.001
PANSS-N pre	18.60 (15.57 to 21.63)	8.11	17.67 (14.26 to 21.07)	9.12			
PANSS-N post	17.50 (14.65 to 20.35)	7.63	13.87 (11.39 to 16.34)	6.63			
PANSS-N change	−1.10 (−2.47 to 0.27)	3.67	−3.80 (−5.85 to −1.75)	5.49	5.169	0.027	0.083

Subsequently follow-up univariate tests (*t*-test for dependent samples) were carried out in ANCOVA’s significant variables confirming that the effect was due to improvement in the intervention group and not to deterioration in the control group (*P* < 0.001). Changes in PANSS negative (*P* = 0.001) and AIHQ aggressive bias (*P* = 0.018) were also due to improvement in the intervention group.

There were no differences in the seven variables of the SFS-SR.

## Discussion

One of the main objectives of cognitive therapy in schizophrenia is to improve social functioning. In this regard, SC programs seem more promising than those directed at neurocognition (29). However, during the last decade, SC rehabilitation has been delivered in group format requiring a significant number of sessions and specialized training for the therapists, therefore limiting its accessibility (64). Bearing these questions in mind, our team designed ET showing its feasibility in a pilot study (32). This study is the first randomized controlled trial conducted with this program.

After treatment, the intervention group showed a significant improvement in ER (Table 2) reaching scores posttreatment within the normal range (65), this result is consistent with other interventions (40, 66–68).

Regarding ToM, the intervention group showed significant improvements at Faux Pas, Happé’s Strange Stories, Hinting Task, and MASC (Table 2). However, even with this improvement, our intervention group did not achieve the level of competence of the healthy population, as was found in other studies (32, 33, 69). Nevertheless, our study indicates that online rehabilitation of complex domains of ToM is possible and also that our training strategies are in the correct path.

Unfortunately, the ANCOVA results in AS only show changes in the Aggressive bias of ambiguous scenes with a reduced effect size. However, this is no surprising because the metacognitive instructions delivered with our ToM short film are not focused on AS and should perhaps deserve a specific module. Nevertheless, the absence of positive results in this domain is consistent with other studies (28, 70).

Furthermore, there were no differences in terms of emotional intelligence assessed with MSCEIT after the intervention, as we can see in Table 2, the pretests in both groups were in the normal range [on the MSCEIT’s IQ-like scale with a mean of 100 and a SD of 15, a respondent would have to get a score higher than 116 or lower than 84 to be statistically significantly (*P* < 0.05) above or below average]; therefore, the instrument seemed unable to detect impairments in ER or ToM nor changes after treatment, a finding also consistent with previous studies (33, 47, 70). For a more detailed review on the concerns over the MSCEIT’s validity, see Maul (2012) (71).

Finally, there was a reduction in PANSS-negative change score (Table 2) in the intervention group, suggesting an eventual effect of the intervention in reducing negative symptoms (3).

In conclusion, *e-Motional Training*^®^ is one of the first online programs that has shown its usefulness in the training of the most studied SC domains. Compared with other available programs (28, 72), this program allows online self-training and follow-up by therapists, thus filling the lack of similar intervention instruments in our community.

Our study has a number of limitations, including the fact that most participants in the sample underwent drug treatment; therefore, we do not know whether the relationships found in this study can be replicated in other populations, including individuals who refuse to undergo treatment. Most of the participants in our sample had a diagnosis of chronic stable schizophrenia; therefore, we ignore the performance and feasibility of ET in first episodes or in individuals at high risk for psychosis. Moreover, the majority of the study participants were men, and therefore, the generalizability of the results must be regarded with caution. However, it is a well-known fact that schizophrenia is more severe in men than in women, and therefore, day hospitals and day centers are more frequented by men than women (73).

Regarding participation remarkably, attendance in our sample was perfect. Although this fact could be surprising, it is worth noting that research studies in schizophrenia in our community are scarce, and therefore, it is easier to raise the interest of patients as well as therapists and evaluators, especially if the active treatment is a computerized online program with an attractive interface, cognitively not demanding and allowing self-training, factors that should be taken into account to explain the adherence of patients during the study.

Regarding our results on social functioning, measured with the SFS-SR, the lack of significance of our findings should be considered in the light of the following facts: given that chronic patients have insight and metacognitive deficits, using a self-evaluated scale to measure social functioning was not the best idea. Moreover, it seems to us that social functioning has to be the goal but probably a standard too high for computerized interventions. This is a common place in other clinical domains, for instance in Alzheimer’s, where generalizability of computerized interventions to daily living is currently absent (74). Our aim is to create an online tool for helping patients to practice ER and ToM interactions but by no means to substitute group therapy or social skills training. In our opinion, computerized tools give the patients the opportunity to drill and practice skills hardly rehearsable outside the virtual realm, but at least in chronic cases, these skills should be trained *in vivo* in protected environments before aspiring to show generalization in the real world.

Finally, the study was conducted vs. TAU and not vs. another active condition. This is obviously not the best design, but our inspiration was based in recent studies in SC rehabilitation both in group therapy and with computerized tools (32, 35, 75–79). However, it must be taken into account that there is a scarcity of data regarding efficacy of computerized programs for SC and that comparing at this point a computerized tool with group strategies seems at least to us unfair.

In terms of the program’s future, version 2.0 is now available, including version 1.0 games and ER tasks devised to improve processing speed, mimicry abilities, and prosodic recognition. Regarding ToM, a short film with real actors and a 2.5 h gameplay graphic adventure with puzzles on ToM and moral dilemmas have been included, and their aim is to offer a gradual and longer training maintaining the attention of patients and their will to improve. The environment has been created with game mechanics, and it has metacognitive hyperlinks designed for self-training.

## Ethics Statement

The study was approved by the Clinical Research Ethics Committee of Galicia (EC) and met all applicable ethical and legal standards (registration code 2014/459) and it has been registered in BioMed Center an international RCT database (ISRCTN83459317).

## Author Contributions

AGC (principal investigator), YMS, and MVC are the creators of e-Motional Training. YMS, MVC, and AGC designed the study, selected participants, applied the intervention, extracted data, and supervised the study. FDL participated in patient selection and in obtaining and extracting data, and MRA and RM reviewed the manuscript. Moreover, RM contributed to perform statistical analysis and writing of the final version. The manuscript was authored by YMS, MVC, and AGC. All the authors have had full access to data in the study, have personally reviewed the manuscript, and gave final approval of the version attached.

## Conflict of Interest Statement

AC, YS, and MC are the intellectual authors of *e-Motional Training*^®^ 1.0, a program funded by the Galician Department through the PRIS program, whose objective is to generate a spin-off.
